# Medial wall orbital decompression surgery for the treatment of Graves’ ophthalmopathy: follow-up results in a single medical center

**DOI:** 10.1007/s10792-025-03635-x

**Published:** 2025-06-25

**Authors:** Meydan Ben Ishai, Daniel Yagoda, Judith Dadon, Tzippy Shochat, Ethan Soudry, Inbal Avisar

**Affiliations:** 1 Ophthalmology Department, Rabin Medical Center, Petach Tikva, Israel; 2 Sackler Faculty of Medicine, Tel Aviv University, Tel Aviv, Israel

**Keywords:** Decompression, TED, Medial wall, Follow-up, Stability

## Abstract

**Purpose:**

To examine the success rate of endoscopic decompression surgery in Graves’ ophthalmopathy.

**Methods:**

This is a retrospective cohort study of all patients who underwent endoscopic decompression surgery at the Rabin Medical Center, Israel, between 2010 and 2022. The data includes post-surgery follow-up time, visual acuity, proptosis outcomes, and post-surgical complications.

**Results:**

Thirty patients underwent unilateral or bilateral decompression surgery at our medical centre during the study period, a total of 42 eyes. The mean age at the time of decompression was 49 ± 15.82 years. The reason for surgery was proptosis in twenty-eight patients (93.3%), while only two patients (6.67%) were referred to surgery due to compressive optic neuropathy. The medial wall was decompressed in all of our patients either as a single procedure or in combination with other procedures. Thirteen patients (43.33%) underwent medial wall-only surgery, thirteen patients (43.33%) had medial and lateral wall surgery, and four patients (13.33%) had medial, lateral, and inferior wall decompression. The mean difference between pre-and post-op of the operated eye, measured by Hertel exophthalmometer, was 3.39 ± 2.45 mm (*P* < 0.001). The mean difference between the operated eye’s visual acuity between pre and post-op was 0.04 ± 0.21LogMAR (*P* = 0.19). The mean follow-up time for the Hertel measurement was 460.10 days (SD = 585.36). We do not report long-term surgical complications.

**Conclusion:**

In the TED (thyroid eye disease) patient, endoscopic medial wall decompression can ensure sufficient orbital decompression while minimising complications. The size of the proptosis should be a factor when planning the surgery.

## Introduction

In TED, the extraocular muscles and orbital fat enlarge due to fibrosis, causing orbital congestion and proptosis. These anatomical changes can result in compressive optic neuropathy, exposure keratopathy, and abnormal ocular motility.

As a result of the discrepancy between swollen tissues and the fixed bony orbit volume, proptosis and orbital congestion occur, leading to swelling of the periorbital membrane and corneal exposure, which can result in compressive optic neuropathy [1].

In TED, orbital decompression resolves rehabilitation issues, including proptosis and scar tissue, correct squints, and repair lids.

Utilising the adjacent sinus spaces, orbital decompression is employed to treat proptosis resulting from the disease. Depending on the patient’s presentation, the decompression required will vary [2, 3].

A vast amount of literature exists regarding orbital decompression for TED, including neurosurgery, head and neck surgery, and maxillofacial surgery. Despite significant technological advancements, four main approaches remain: transorbital, transcranial, transantral, and transnasal [4, 5].

Various types of orbital decompression have been described, including single-wall, double-wall, and triple-wall bony decompression, as well as solely fatty decompression with the endoscope [6].

The most effective intervention, however, is not agreed upon. Surgical techniques vary geographically and are largely determined by the preferences of surgeons [7].

Decompression can be achieved via different areas, including the floor, the medial wall, the inferomedial wall, the lateral wall, and the fat compartment. The literature reveals no significant difference among the transconjunctival, transantral, and transcutaneous approaches in terms of overall surgical outcomes [5, 8].

Today, the trans-nasal endoscopic method is a common procedure that appears to reduce proptosis and improve visual acuity safely and effectively [9–11].

Multiple publications have reported varying results regarding the surgical method, the number of orbital walls affected, and the amount of orbital fat removed [12–16].

Furthermore, numerous studies have reported long-term outcomes following orbital decompression surgery, including proptosis and visual acuity changes and complications [17–21].

This study aims to describe the results of orbital decompression surgery in our medical centre.

## Methods

A retrospective case series of endoscopic orbital decompression was conducted at Rabin Medical Centre, Israel, between 2010 and 2022 by a single surgeon (IA). All cases of endoscopic decompression for TED were included in this paper with no exclusions. The endoscopic decompression technique was well described in previous papers [11] and will not be discussed in this paper.

The statistical analysis for this paper was generated using SAS software, version 9.4.

Continuous variables were presented by mean ± SD, and categorical variables were presented by (N, %).

The normality of continuous variables was assessed using the Kolmogorov–Smirnov test.

A linear mixed model, with repeated measures for subjects who underwent surgery on both eyes, was used to compare post-operative to pre-operative values and differences by wall.

Two-sided p-values less than 0.05 were considered statistically significant.

The primary outcomes evaluated were changes in proptosis, as measured by Hertel exophthalmometer, and visual acuity. The initial post-operative Hertel and visual acuity measurements were taken at the first post-surgical clinic follow-up, typically within 1–3 months after surgery. Long-term surgical stability was assessed using the latest available Hertel measurements, obtained during routine follow-up visits.

## Results

**Fig. 1:**
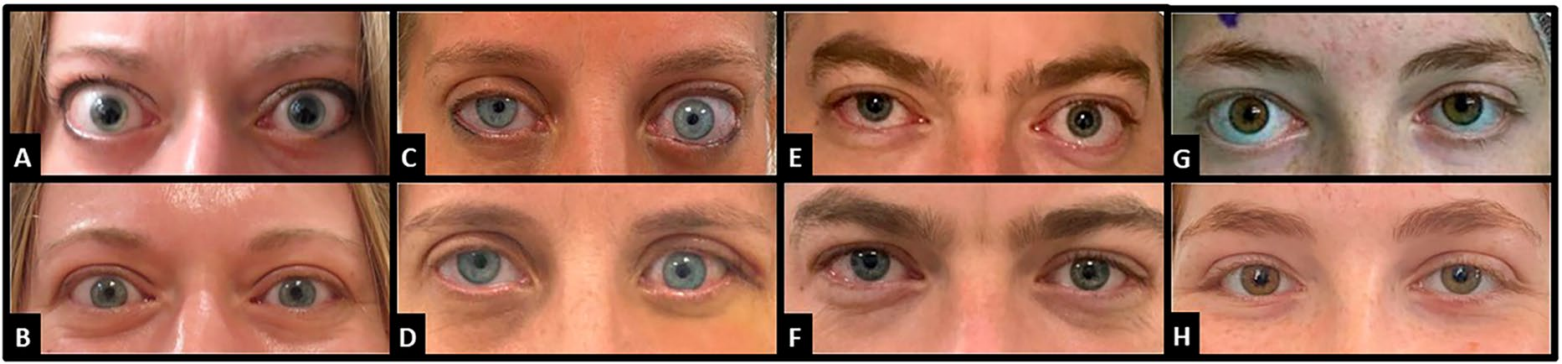
Pre and post-operative photos of four patients. **A**, **B** Patient 1 (46F, BE medial wall only). **C**, **D** Patient 5 (43F, LE medial and lateral walls). **E**, **F** Patient 16 (42M, BE medial and lateral walls). **G**, **H** Patient 27 (18F, RE*, Medial and lateral walls). *BE* Both eyes, *LE* Left Eye, *RE* Right Eye

**Fig. 2:**
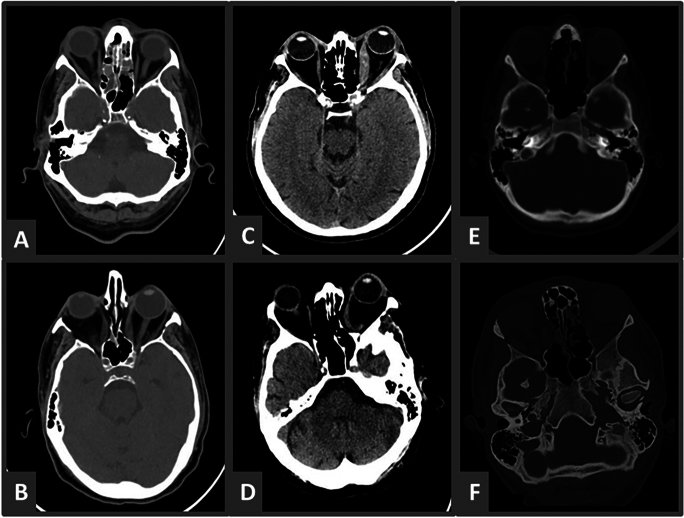
Pre and post-operative axial CT scans. **A**, **B** Patients 17 (55M, BE medial and lateral walls). **C**, **D** Patient 2 (61M, LE medial wall only). **E**, **F** Patient 27 (18F, RE, Medial and lateral walls)

Our study group consisted of 30 patients (36.67% male). Eighteen patients (60%) underwent unilateral decompression, and twelve (40%) underwent bilateral decompression—42 eyes. The mean age at decompression was 49 ± 15.82 years, and eleven (36.67%) were smokers. (Table 1, Figs. 1, 2).

**Table 1:** Patients characteristics and measurements before and after surgery

Case number	Age at time of SX	Sex	Indication for surgery	Pre-Op treatment	Eye	Walls decompressed	Best correct visual aquity (LogMAR) of operated eye		Exophthalmometry (mm) of operated eye	
							Pre op	Post op	Pre op	Post op
1	46	F	Proptosis	N/A	Both	Medial only	0.07/0.07	0.07/0.07	20/23	19/20
2	61	M	Compressive optic neuropathy	Merkaptizol	Left	Medial only	0.12	0.12	23	22
3	71	F	Proptosis	Euthyrox, Iodine	Both	Medial,Lateral,Inferior	0.12/0.07	0/0.07	28/29	22/23
4	89	F	Compressive optic neuropathy	Merkaptizol	Left	Medial only	1.3	0.07	24	17
5	43	F	Proptosis	Merkaptizol, Botox	Left	Medial, Lateral	0	0	22	17
6	37	F	Proptosis	N/A	Right	Medial,Lateral,Inferior	0	0	26	21.5
7	49	F	Proptosis	Steroids, Merkaptizol, Mycophenolate	Both	Medial only	0/0	0/0	22/24	20/20
8	52	F	Proptosis	Euthyrox, Thyroidectomy	Both	Medial only	0.07/0.07	0.07/0.07	19/20.5	17/18
9	18	F	Proptosis	Merkaptizol	Right	Medial only	0	0	24	21
10	23	M	Proptosis	Merkaptizol, Iodine	Both	Medial, Lateral	0/0	0/0	27/22	21/21
11	49	F	Proptosis	Steroids	Right	Medial, Lateral	0.07	0	30/30	25/28
12	68	F	Proptosis	Eltroxin, Thyroidectomy	Left	Medial, Lateral	0.12	0.3	23	19
13	62	M	Proptosis	Steroids, Radiation	Both	Medial only	0.17/0.17	0.12/0.17	29/28	26/25
14	40	F	Proptosis	None	Right	Medial only	0	0	23	20
15	38	M	Proptosis	Botox	Right	Medial only	0.12	0	23	19
16	42	M	Proptosis	PTU (Propylthiouracil)	Both	Medial, Lateral	0.12/0.12	0/0	30/27	25/24
17	55	M	Proptosis	Steroids, Merkaptizol	Both	Medial, Lateral	0/0	0/0	29/27	28/26
18	47	F	Proptosis	Iodine	Both	Medial, Lateral	0.18/0.07	0.18/0.18	24/23	22/23
19	61	M	Proptosis	None	Left	Medial, Lateral	0.07	0.07	24	27
20	36	M	Proptosis	Merkaptizol, Botox	Both	Medial only	0.12/0.07	0 /0.07	28.5/31.5	27/27
21	65	F	Proptosis	Euthyrox, Thyroidectomy	Right	Medial only	0	0.07	25	23
22	56	M	Proptosis	N/A	Both	Medial,Lateral,Inferior	0/0	0/0	30/30	26/27
23	70	M	Proptosis	Steroids, Radiation, Euthyrox, Thyroidectomy	Both	Medial, Lateral	0.4/0	0.07/0.07	28/27	20/25
24	39	F	Proptosis	Euthyrox, Merkaptizol, PTU	Right	Medial, Lateral	0	0	26	18
25	41	F	Proptosis	PTU	Left	Medial, Lateral	0.13	0	24	20
26	42	F	Proptosis	N/A	Left	Medial only	0	0	22	19
27	18	F	Proptosis	Steroids, Merkaptizol	Right	Medial, Lateral	0	0	22	19
28	46	F	Proptosis	Euthyrox, Iodine	Left	Medial, Lateral	0	0	22	19
29	46	F	Proptosis	Euthyrox	Left	Medial only	0	0	23	18
30	60	M	Proptosis	Steroids, Radiation	Left	Medial,Lateral,Inferior	0.13	0	30	23

Regarding pre-operative medical treatment, data was available for 26 (86.67%) patients only. Most of those patients (92.33%) received some form of medication (Table 1), while only two (7.69%) were not treated at all.

The reason for surgery was proptosis in 28 patients (93.3%), while only two patients (6.67%) were referred to surgery due to compressive optic neuropathy.

The medial wall was decompressed in all our patients either as a single procedure or in combination with other procedures. Thirteen patients (43.33%) underwent medial wall-only surgery, 13 patients (43.33%) had medial and lateral wall surgery, and four patients (13.33%) had medial, lateral, and inferior wall decompression.

The initial post-operative Hertel measurement was obtained at a median time of approximately 1–3 months after surgery, with a mean pre-operative value of 25.8 ± 3.58 mm and a mean post-operative value of 21.69 ± 3.29 mm, yielding a statistically significant mean reduction of 3.39 ± 2.45 mm (*P* < 0.001).

A total of 17 eyes (40%) had long-term follow-up data available for surgical stability assessment, with a mean interval of 1.75 years (SD = 1.52) between the initial post-operative and the latest Hertel measurements. A mean deterioration of 0.58 mm (SD = 1.23) was observed over this period. The mean difference between the operated eyes’ visual acuity between pre and post-op was 0.04 ± 0.21LogMAR (*P* = 0.19).

Comparing Hertel measurements among the three groups—medial wall only, medial and lateral walls, and medial, lateral, and inferior walls, the mean difference between pre-and post-operative values was 2.35, 3.18, and 4.70 mm, respectively. Nevertheless, we did not find those differences statistically significant. The mean follow-up time for the Hertel measurement was 460.10 days (SD = 585.36).

There were no short or long-term surgical complications in any of our patients. Despite this, deterioration is expected following decompression surgery and is not considered a complication.

Over a mean period of 1.75 years (SD = 1.52), surgical results deteriorated to a mean of 0.58 mm (SD = 1.23). This was determined by comparing the Hertel measurement obtained after surgery with the most recent Hertel measurement obtained for each patient. This data, however, was only available for 17 eyes (40%).

## Discussion

In this study, the authors report a retrospective case series of endoscopic orbital decompression surgery for proptosis caused by TED. The results of endoscopic decompression are comparable to those reported in the literature.

The post-operative results in our study demonstrate significant improvement in proptosis within the early post-operative period (typically measured within 1–3 months after surgery). Importantly, we also assessed the long-term stability of these results over a mean duration of 1.75 years using repeated Hertel measurements.

The authors report an average decompression of 2.35 mm from medial wall-only surgery, 3.18 mm from surgery involving both medial and lateral walls, and 4.7 mm from triple-wall surgery. Those results are comparable to 2.6–6 mm improvement with endoscopic approaches and 3.2–6.5 mm with external approaches reported in the literature today. The average reduction of proptosis in the 3-wall decompression group was 7.6 mm [1, 4–17, 19–30].

The authors’ results showed good, statistically significant differences in all three methods (medial alone, inferomedial, and lateral combined with inferomedial). There was a difference in the proptosis improvement (2.35, 3.18, and 4.70 mm, respectively), although this was not statistically significant. This is likely due to the low power of the small sample size.

Results vary across the literature regarding visual acuity, probably due to the heterogeneity of patients and surgical indications [20, 31]. The visual acuity in this study remained stable in all cases. This is due to good visual acuity pre-operation in most patients (0.01 ± 0.21LogMAR) combined with a small study group. It is also known, from recent publications, that visual acuity has a better chance of improving in the active phase of the disease and in patients with compressive optic neuropathy than in those with long standing proptosis [22, 32].

Regarding common complications, endoscopic decompression has been associated with diplopia occurring at a rate of 15–45% [6, 9, 11, 15, 20, 22, 25, 28, 31]. No long-term complications were reported in this series, probably because the study group was small.

In our study, we noted a wider range of proptosis reduction in the medial wall-only decompression group compared to the multi-wall groups. We hypothesise that this variability may be due to patient-specific anatomical factors. For instance, the degree of ethmoid sinus pneumatisation can vary significantly, directly impacting the volume available for medial orbital decompression. Additionally, individual differences in orbital fat volume and distribution, particularly adjacent to the medial wall, may impact the effectiveness of decompression. Tissue compliance and the extent of fibrosis resulting from prior inflammation may also affect the amount of orbital content that can prolapse into the ethmoid space. Further radiological or volumetric studies correlating sinus anatomy and orbital tissue characteristics with surgical outcomes may help clarify these observations.

An interesting finding in our study was the slight deterioration in Hertel measurements over time, with a mean increase of 0.58 mm occurring approximately 1.75 years after surgery. Although this change is small, it raises questions regarding long-term surgical stability. We propose several possible explanations for this finding:

First, subclinical progression of TED may occur even during the quiescent phase, potentially leading to minor changes in orbital tissue volume or stiffness. Second, age-related remodelling of orbital fat and connective tissue over time may alter the positioning of the globe, independent of disease activity. Third, tissue elasticity and mechanical rebound may contribute to a mild re-expansion or settling of orbital contents following decompression. Finally, it is essential to acknowledge that Hertel exophthalmometer, although widely used, is susceptible to inter- and intra-observer variability, particularly in long-term follow-up, where measurements may not be taken under identical conditions.

This study has some limitations. Since clinical data were collected retrospectively, they lack unity and may be insufficiently documented. Secondly, the number of patients in the current study may be insufficient to yield significant results for some questions. Only 56% of the study patients underwent a third Hertel measurement, so the deterioration data was limited. It may be necessary to conduct larger multicenter prospective comparative studies to obtain sufficient data and more conclusive results. In addition, this study represents a retrospective series of endoscopic decompressions and, therefore, cannot be used to compare the outcome with that of external approaches. Smoking is a well-known risk factor for TED severity and poorer outcomes with both medical and surgical treatments. Although we recorded smoking status in our cohort, we did not perform a formal comparison of surgical results between smokers and non-smokers. Additionally, the retrospective nature of our data limited the consistency and detail of smoking history documentation, such as pack years and cessation status. Future studies should explore the impact of smoking on surgical outcomes in TED, ideally with prospective data collection and standardised definitions of smoking exposure. Furthermore, although data on pre-operative medical therapy were available for most patients in our study, the variability in treatment type and timing, along with our limited sample size, prevented a formal analysis of its impact on post-operative outcomes. Future prospective studies are needed to determine whether certain pre-operative therapies influence surgical results, particularly regarding inflammation control, tissue compliance, and healing response.

## Conclusion

Endoscopic medial wall decompression is a safe and effective treatment for proptosis in thyroid eye disease, offering significant short-term improvement and stable long-term outcomes with minimal complications. While visual acuity remained largely unchanged, the procedure consistently reduced proptosis across surgical variations. Further large-scale studies are needed to confirm these findings and clarify the impact of individual patient factors on surgical results

## Data Availability

No datasets were generated or analysed during the current study.
